# Single-shot Stokes polarimetry of plasmon-coupled single-molecule fluorescence

**DOI:** 10.1515/nanoph-2025-0352

**Published:** 2025-11-06

**Authors:** Sarojini Mahajan, Yuyang Wang, Teun A.P.M. Huijben, Rodolphe Marie, Peter Zijlstra

**Affiliations:** Department of Applied Physics and Science Education, and Institute for Complex Molecular Systems, 3169 Eindhoven University of Technology, 5600 MB Eindhoven, The Netherlands; Department of Health Technology, Technical University of Denmark (DTU), 2800 Kongens Lyngby, Denmark

**Keywords:** plasmon resonance, single-molecule fluorescence, single-molecule polarimetry, single-particle spectroscopy, plasmon-enhanced fluorescence

## Abstract

The photophysical properties of single-molecule emitters are altered by nanophotonic structures such as single plasmonic nanoparticles. The intensity and spectral properties of plasmon-coupled emitters have been studied extensively, but little is known about the effect of plasmon coupling on emission polarization. Here, we examine how particle-emitter coupling modifies the polarization of single fluorophores in both experiment and simulation. We quantify degree of linear polarization using Stokes polarimetry with a polarization-sensitive camera and quantify the Stokes parameters with a single-shot acquisition without requiring additional optics in the detection path. We then perform polarization-resolved measurements of plasmon-coupled fluorescence from single-molecule emitters using an approach based on DNA-PAINT. We quantify the effect of the setup and associated noise sources on the measured Stokes parameters. We then quantify the angle of linear polarization (AoLP) and the degree of linear polarization (DoLP) for thousands of single molecules. We compare our results to a numerical model that propagates the plasmon-coupled single-molecule emission through the optical setup to yield the polarized point spread function in the camera plane. Simulations and experiments are in good agreement and shed new light on the polarization of antenna-coupled fluorophores, while it establishes single-shot polarimetry as a promising and straightforward method to quantify polarization properties at the single-molecule level.

## 1 Introduction

Understanding fluorescence emission from a single fluorescent emitter has been crucial for studying complex light–matter interactions at the nanoscale [1], [2], [3]. Optical properties become intriguing when single fluorescent emitters are placed near metallic nanoparticles. A metallic nanoparticle acts as an antenna that modifies the properties of a single emitter, such as its emission intensity [4], [5], [6], [7], [8], [9], spectrum [10], [11], [12], radiation pattern [13], [14], [15], and directivity [16], [17], [18], [19]. Plasmon-coupled single molecules, therefore, serve as a fascinating model system to investigate light–matter interactions, which is crucial for the advancement of applications in sensing, microscopy, spectroscopy, and photonics.

Standard optical methods, including wide-field and confocal microscopy, are widely employed to investigate the effect of plasmon resonances on single-molecule fluorescence. Enhancement of fluorescence brightness results from near-field interactions, which lead to changes in emission intensity, lifetime, and spectral characteristics [5], [20]. However, in a typical wide-field camera-based approach, polarization-resolved information is not directly accessible, since the camera itself does not separate polarization states.

Nonetheless, it is possible to extract information about polarized emission and emitter orientation by analyzing the shape of the point spread function (PSF) or using defocused imaging. One approach to determine a single emitter’s 3D orientation and location requires PSF engineering and subsequent fitting. This method relies on complex setups and analysis to determine molecular orientation [21], [22], [23]. Other techniques, such as defocused imaging [24], [25], [26] or back focal plane imaging [27], [28], also require intricate analysis and are often limited by signal-to-noise ratio [29], [30]. Hübner et al. [26] used the defocused imaging method to demonstrate that DNA origami-assembled optical antennas made of gold nanoparticles can direct the emission direction of a freely rotating fluorophore. They also examined excitation directionality using polarization-resolved excitation measurements. A straightforward alternative to determine the orientation of an emitter is to modulate the polarization of the excitation beam. The emitter absorbs light most effectively when the polarization aligns with its dipole moment, enabling us to deduce its orientation [31], [32]. This allows the quantification of the emitter’s angle within the sample plane. However, it is possible to extract both the in-plane (*α*) and out-of-plane (*β*) dipolar angles of the emitter by changing the illumination strategy [30], [33]. Ming et al. [34] measured the dependence of plasmon-enhanced fluorescence on polarization using excitation polarization modulation. Another widely used method to study the polarization of emitted light from plasmon-emitter coupled systems is polarization splitting. This technique involves using a polarization beam splitter to separate the emitted light into two or four polarization components. By analyzing the intensity ratio of these components, information about the molecular orientation can be extracted. Several research groups have employed this approach to determine the emission polarization of single molecules in proximity to plasmonic nanoparticles [35], [36], [37]. These studies measure only two orthogonal polarizations, which is insufficient to quantify all four Stokes parameters. The full set of Stokes parameters can be resolved when four polarization components are measured, as demonstrated by Ohmachi et al. [38] and Rimoli et al. [39] by splitting the detection path into four polarization channels to capture the full polarization state and orientation of single emitters.

Here we present a straightforward method that, without the need of added optical elements, captures four polarization states in a single shot and thereby enables the quantification of all four Stokes parameters. The method is based on a polarization camera and enables wide-field polarization-resolved imaging with single-molecule sensitivity. We employ this approach to investigate single-molecule fluorescence polarization by coupling single emitters to plasmonic nanoparticles using an approach based on DNA-point accumulation for nanoscale topography (DNA-PAINT). We compare experiments to simulations to quantify the degree of linear polarization (DoLP) of single fluorophores coupled to gold nanospheres and nanorods. Our results show that the emission from freely rotating single emitters is significantly polarized by coupling to both isotropic and anisotropic plasmonic structures. Coupling single emitters to spherical nanoparticles results in an effective linear emission dipole whose orientation is determined by the emitter’s binding site on the sphere. In contrast, for anisotropic structures such as nanorods, the emission dipole tends to align along the long axis of the particle. This approach enables detailed investigations of nanoscale polarization dynamics and creates new possibilities for studying plasmon-enhanced emission in complex environments.

## 2 Results and discussion

**Figure 1:**
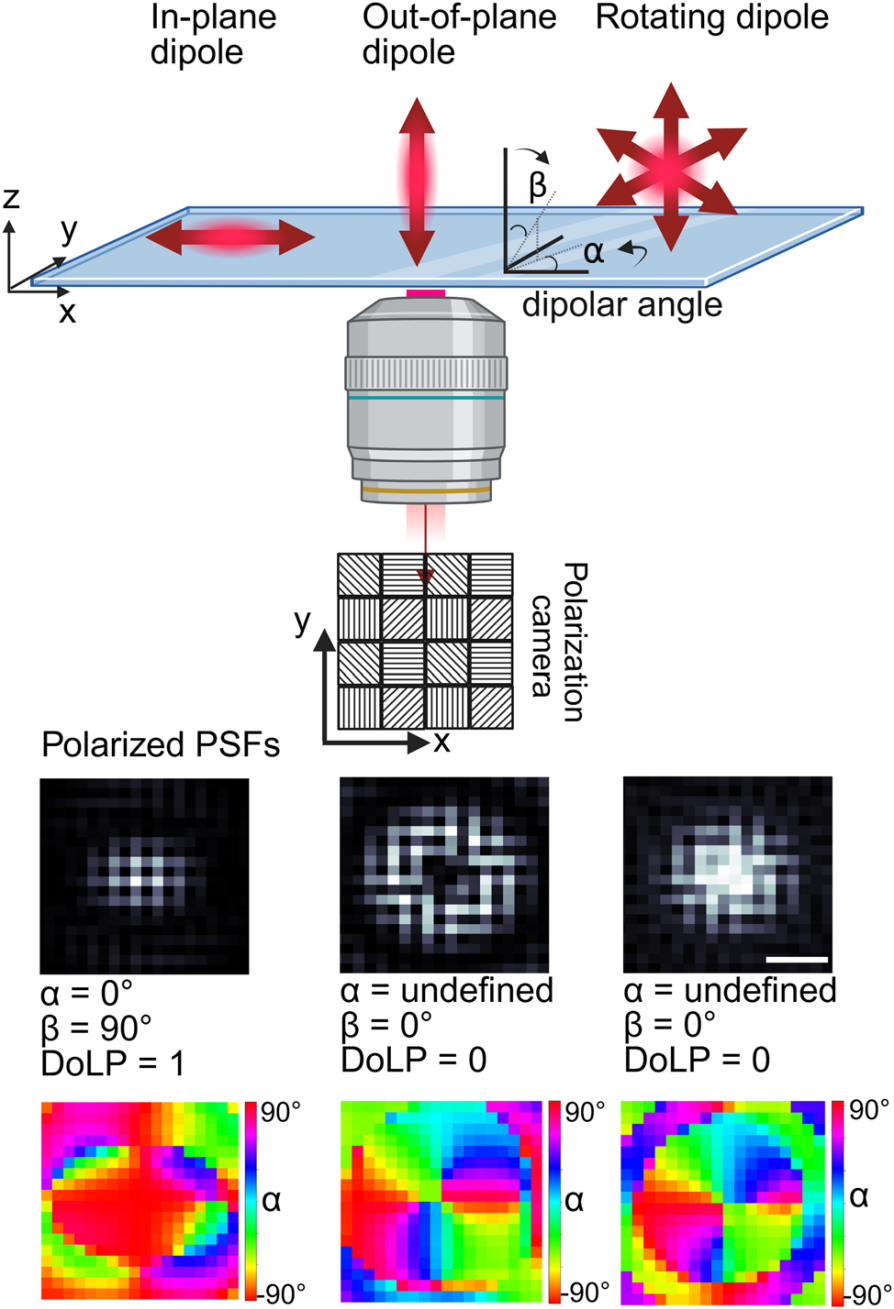
Schematic representation of the polarization detection for dipole emitters. The top diagram illustrates in-plane, out-of-plane, and rotating dipole orientations relative to the imaging system. The polarization camera captures intensity distributions through a 2 × 2 micropolarizer array with distinct transmission axes, as shown in the pixel scheme. The bottom row presents simulated point spread functions (PSFs) for different dipole configurations. The bottom row shows the corresponding polarization color map of the AoLP for the same dipole emitters in the middle row. The scale bar (200 nm) applies to all PSFs.

To enable quantification of all Stokes parameters in a single image we employ a polarization camera that was developed for machine vision applications. More recently, Bruggemann et al. [40] used a polarization camera for single-molecule orientation localization microscopy and established the method to instantly determine molecular orientations, focusing primarily on applications in life sciences. The polarization camera is a regular CMOS camera with a grid of polarizers in front of the chip. The grid of polarizers creates groups of 4 pixels (2 × 2) that resolve polarization angles at 0°, 45°, 90°, and 135° as shown in Figure 1. The Stokes parameters are then calculated for each group of four pixels and are defined as follows:

(1) $$S_{0}=\left(I_{135{}^{\circ}}+I_{0{}^{\circ}}+I_{90{}^{\circ}}+I_{45{}^{\circ}}\right)/2,$$

(2) $$S_{1}=I_{0{}^{\circ}}-I_{90{}^{\circ}},\;\text{and}$$

(3) $$S_{2}=I_{45{}^{\circ}}-I_{135{}^{\circ}},$$

where *I*_angle_ indicates the number of photons reported by the respective pixel. The orientation of a fixed emitter is determined from the intensities measured in each detection channel of the polarized camera, as derived by Fourkas [41]. The in-plane angle (*α*) and out-of-plane angle (*β*) can be calculated using the Stokes parameters in the following way:

(4) $$\alpha =\frac{1}{2}\tan^{-1}\left(\frac{S_{2}}{S_{1}}\right),\text{and}$$

(5) $$\beta =\sin^{-1}\left(\sqrt{\frac{A\cdot \text{DoLP}}{C-B\cdot \text{DoLP}}}\right),$$

where A, B, and C are constants that depend on the collection angles of the objective lens and have been defined by Bruggeman [40]. The DoLP is defined as the degree of linear polarization calculated using the average values of the Stokes parameters ⟨*S*_0_⟩, ⟨*S*_1_⟩ and ⟨*S*_2_⟩:

(6) $$\text{DoLP}=\sqrt{\frac{{\left\langle S_{1}\right\rangle }^{2}+{\left\langle S_{2}\right\rangle }^{2}}{{\left\langle S_{0}\right\rangle }^{2}}}.$$

To calculate the AoLP and DoLP of a single-molecule emission event we average the above calculated values over a 20 × 20 pixel region of interest with the PSF centered in it. As such the local polarization structure of the PSF as shown in Figure 1 is taken into account because it affects the average AoLP and DoLP in both the simulations and experiments. To quantify the orientation angle of isolated dye molecules (without plasmonic particle), we directly extract the angles *α* and *β* as defined above. For dye molecules near a plasmonic particle, we find an apparent in-plane and out-of-plane angle *α*_apparent_ and *β*_apparent_. We use this definition to clarify that it is not the dipole that is fixed in orientation but rather that the dye-particle complex exhibits a PSF that represents a near-fixed dipole under angles *α*_apparent_ and *β*_apparent_.

To understand the physics of particle-fluorophore coupling and its effect on emission polarization, we first simulated the point spread function on the polarization camera of a fixed dipole emitter (emission at 675 nm) with various orientations on a glass substrate using the finite-difference time-domain (FDTD) method. The resulting electric fields were then propagated through free space and the imaging system (see Methods) before being projected onto the camera plane. After calculating the polarized images, we calculated the angle of linear polarization (AoLP, quantified by the in-plane angle (*α*) and the out-of-plane dipole angle (*β*)), as well as the degree of linear polarization (DoLP) using the approach described in the Methods section. An example of the simulated camera images of an in-plane dipole emitter, an out-of-plane dipole emitter, and a freely rotating dipole are depicted in Figure 1 with the extracted values for *α*, *β*, and DoLP that follow from the simulated PSF.

**Figure 2:**
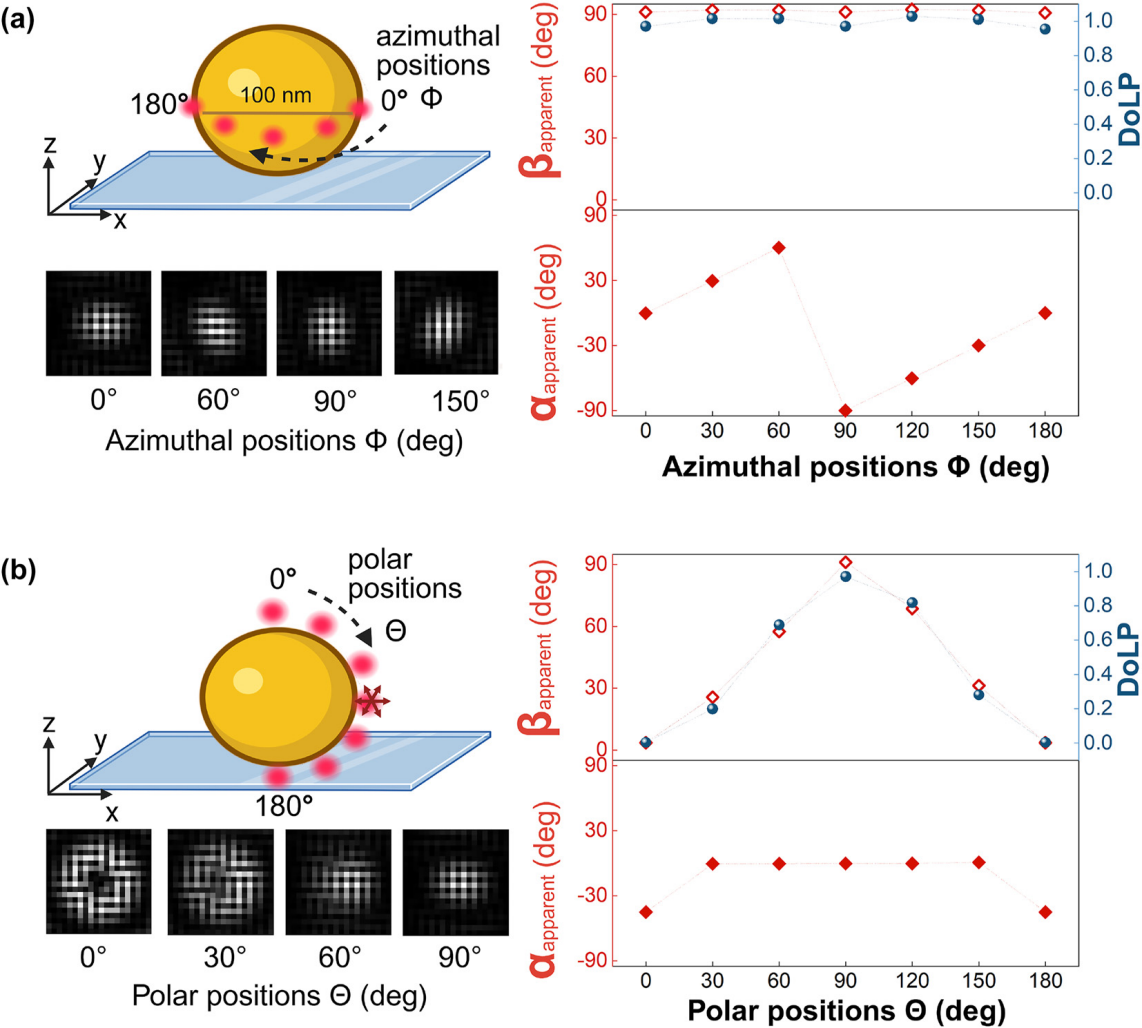
Simulated point spread functions on the polarization camera for the emission of a freely rotating dipole emitter (emission at 675 nm) near a gold nanosphere in water, captured with a 1.49 NA oil immersion objective lens. (a) Simulated point spread functions for dipoles positioned at different azimuthal angles (*ϕ*) around the equator of a 100 nm gold nanosphere on a glass substrate. The in-plane polarization angle (*α*_apparent_) follows *ϕ*, while the out-of-plane angle (*β*_apparent_) remains approximately 90°. The degree of linear polarization (DoLP) is consistently high. (b) Simulated point spread functions for dipoles placed at different polar angles (*θ*) from the north to south pole of the nanosphere. Here, *β*_apparent_ aligns with *θ*, while *α*_apparent_ remains 0° except at the poles, where it is undefined. The DoLP varies between 0 and 1, reflecting the transition from out-of-plane to in-plane emission at the equator. The scale bar (200 nm) applies to all PSFs, while dotted lines show the expected values.

In the case of a fixed emitter, the detected emission is polarized along the dipole axis. For an in-plane fixed emitter, the extracted dipolar angle, *α* = 0° while *β* = 90° with a high degree of linear polarization (DoLP = 1). In contrast, for both out-of-plane and freely rotating dipoles, DoLP is 0, and *α* is undefined because all in-plane polarizations are present in the PSF [21]. Figure 1 shows that the out-of-plane dipole and the rotating dipole share the same value of *α*, *β*, and DoLP, making them indistinguishable using polarization alone. However, the PSF shapes differ because the objective lens captures a different radiation pattern for each scenario [14]. The shape of the PSF thereby allows discrimination between unpolarized light from a freely rotating dipole and that from a fixed emitter aligned along the *z*-axis. Supplementary Figure S1 shows the polarized emission from fixed dipole emitters on a glass substrate. After validating our simulation method using this setup, we extended the analysis to study a freely rotating dipole placed at various positions near a gold nanosphere (Figure 2).

The emitter is attached via a flexible linker to the ssDNA probe, so we assume that the emitter’s rotational diffusion is faster than its fluorescence rate. To simulate a freely rotating emitter with an emission at 675 nm, we averaged the electric field contribution from three orthogonal dipoles with equal weights. Figure 2 presents simulated results showing that a freely rotating dipole emitter placed near the surface of a gold nanosphere exhibits polarization behavior different from that when placed near a glass substrate. When the emitter interacts with the gold nanosphere, the local electromagnetic environment constrains the emission such that it resembles that of a fixed dipole aligned with the particle’s surface normal. This effect arises from near-field coupling between the emitter and the metallic surface, which selectively strengthens the dipole normal to the particle surface, whereas emission from the other orientations is suppressed because the fluorophore’s dipole and the induced dipole in the particle are anti-parallel and therefore largely cancel each other. Although this approximation does not fully capture all aspects of the coupled system, it effectively describes the dominant emission direction and polarization characteristics induced by the plasmonic structure.

When the dipole is positioned along the azimuthal angles (*ϕ*) around the equator of the nanosphere, as shown in Figure 2a, the in-plane angle(*α*_apparent_) correlates strongly with the dye’s azimuthal location. In contrast, the out-of-plane angle (*β*_apparent_) remains constant at 90° for all azimuthal positions, indicating that the emission axis lies entirely in the x–y plane. The degree of linear polarization (DoLP) at these positions exceeds 0.9, showing a strongly polarized emission that closely resembles that of a fixed isolated dipole as shown in Figure 1. This trend closely follows the behavior of *β*_apparent_, as both parameters are interrelated through Equation (5).

In Figure 2b, we consider the case where the freely rotating dipole emitter is placed along the polar coordinates (*θ*) near the sphere. Similar to the azimuthal case, the emission axis remains effectively aligned with the surface normal, and the freely rotating emitter continues to behave like a fixed dipole. This is apparent when comparing the emission of the freely rotation dipole (Figure 2b) with the polarized emission of a fixed dipole perpendicular to the sphere’s surface (Figure S2, orientation 1). At the equator, *β*_apparent_ reaches 90°, consistent with in-plane emission. The in-plane angle *α*_apparent_ remains zero across all polar positions, except at the poles (0° and 180°), where *α*_apparent_ becomes undefined due to the symmetry of the geometry. The DoLP in this case varies between 0 and 1, depending on the dye position relative to the particle’s equator. These results highlight how near-field coupling with a metallic nanoparticle can constrain the emission from a freely rotating dipole, thereby changing the polarization to a specific angle that is encoded by the fluorophore’s location. Note that we perform all simulations at a fixed emitter wavelength of 675 nm, because we find that the polychromaticity of the emission only has a minor effect on the polarization response (see Figure S3).

Faithful extraction of polarization properties from an experiment strongly depends on the design of the optical setup to minimize polarization changes induced by optical elements. At the same time, detection noise is inevitably present in any single-molecule experiment due to the low detected intensity. We now analyze both aspects, starting with the effect of detection noise on the extraction of Stokes parameters. The above simulations were conducted assuming noise-free imaging and thereby represent results in the limit of infinitely strong emission intensity. To consider the limited signal-to-noise ratio typically encountered in single-molecule studies, we also simulated the effect of noise on the polarization analysis. The total noise *σ*_total_ in an imaging system can be expressed as:

(7) $$\sigma_{\text{total}}=\sqrt{\sigma_{\text{shot}}^{2}+\sigma_{\text{readout}}^{2}+\sigma_{\text{dark}}^{2}},$$

where *σ*_shot_ represents shot noise, which follows a Poisson distribution due to the stochastic nature of photon detection. The term *σ*_readout_ corresponds to readout noise, which is introduced during the process of pixel readout by the camera electronics and is independent of integration time. Meanwhile, *σ*_dark_ refers to dark noise, caused by thermally generated electrons (dark current) whose contribution depends on exposure time and temperature. Both *σ*_readout_ and *σ*_dark_ are typically modeled as Gaussian noise. Combined, these are referred to as camera noise, denoted by *σ*_camera_. With this simplification, the total noise expression becomes:

(8) $$\sigma_{\text{total}}=\sqrt{\sigma_{\text{shot}}^{2}+\sigma_{\text{camera}}^{2}},$$

which was used to add noise to the simulated PSFs and to quantify the impact of SNR on the analysis. The results, shown in Supplementary Figure S4, highlight the importance of having an SNR > 10 for reliable single-molecule polarimetry. In the remainder of this report, we therefore discard any single-molecule events with an SNR <10. Also, since DoLP and *β*_apparent_ exhibit similar trends, we exclude *β*_apparent_ from further data visualization. In the future, development of lower noise camera’s by e.g. thermoelectric cooling of the camera chip will enable quantification of dimmer events.

Before discussing the experiments, we analyzed the effect of the setup design on the extraction of faithful polarization parameters. To do so, we quantified the polarization distortion caused by the setup alone. We used a wide-field fluorescence microscope for single-molecule DNA-PAINT experiments, which consists of a regular inverted microscope with an excitation laser, a dichroic mirror, an objective lens, a tube lens, and various filters, as shown in Figure S4. However, optical elements in the setup, such as dichroic mirrors, lenses, and other components, can introduce polarization artifacts [42], which we quantified in the polarization distortion effects of the dichroic mirror section of the Supplementary Information. To mitigate these effects in subsequent experiments, we used a 50:50 beam splitter along with an additional emission filter to suppress residual excitation light.

**Figure 3:**
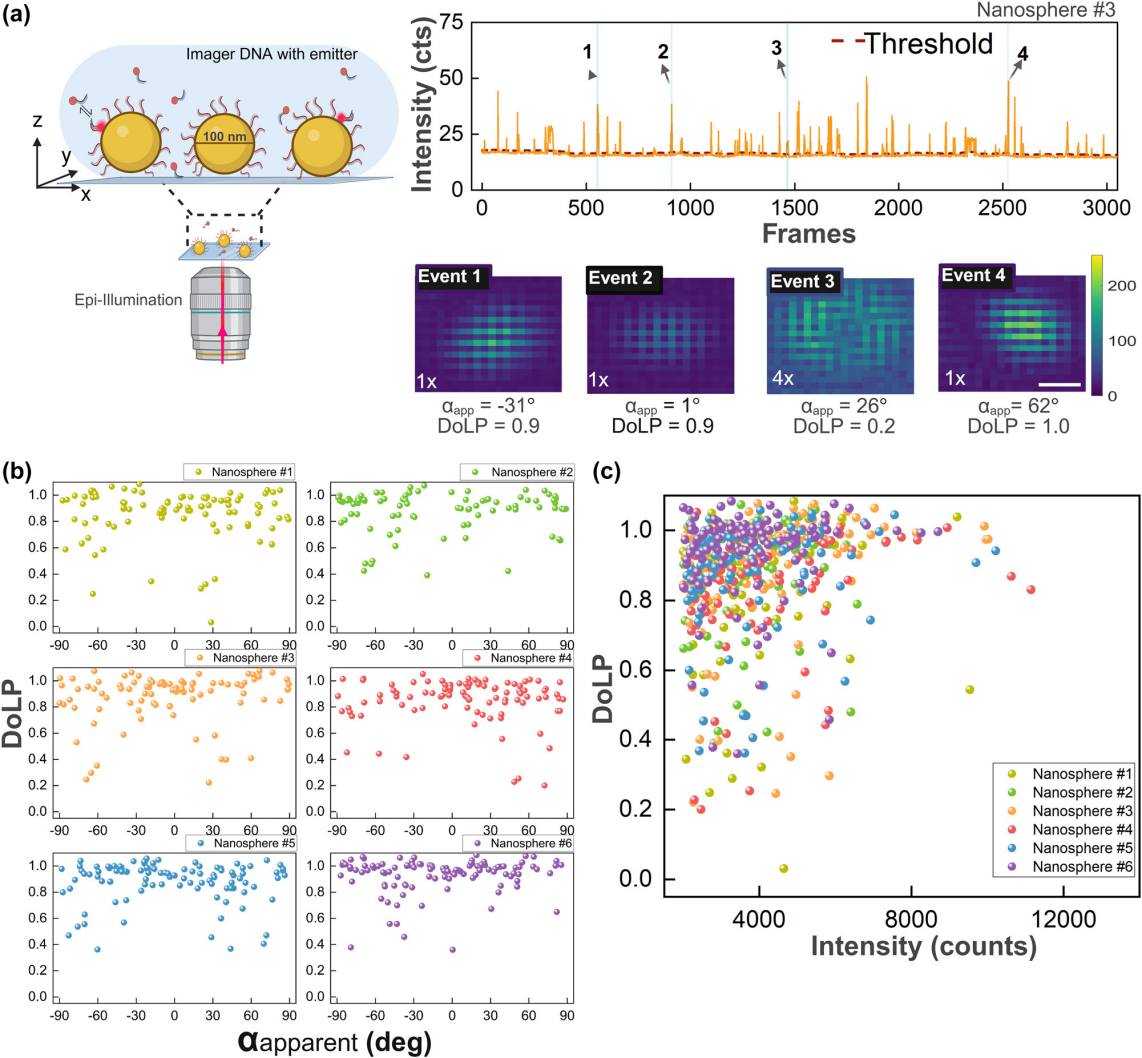
DNA-PAINT experiment on single gold nanospheres. (a) Schematic of a gold nanosphere sample on a glass substrate excited with an epi-illumination fluorescence microscope. Fluorescent intensity time traces are recorded at 10 frames per second, showing bursts with an event typically lasting for 10 frames. Each fluorescent event, averaged over all frames above the threshold, provides a polarized PSF image. Four example events with the extracted *α* and DoLP indicate single-emitter binding to a particle at different locations. Scale bar of 200 nm applies to all PSFs. (b) Scatter plot of DoLP and *α*_apparent_ for fluorescence events on six single nanospheres. (c) DoLP as a function of integrated fluorescence intensity showing that brighter events exhibit a higher DoLP because they occur at the equator of the particle.

We then applied the method to quantify the angle and degree of linear polarization of plasmon-enhanced single-molecule fluorescence. To do so, we performed DNA-PAINT [43] on single nanospheres and nanorods. This approach exploits the reversible binding between single fluorescent probes and ssDNA functionalized nanoparticles. Binding events are stochastic in nature, enabling us to sample many individual binding events across dozens of particles in parallel in a single experiment. In Figure 3a the experimental results are shown for nanospheres functionalized with DNA docking strands and imaged using circularly polarized epi-illumination. Fluorescence timetraces exhibit bright fluorescence bursts on the polarization camera due to binding of the fluorescent probe to docking strands on the particle.

Emission events contain a mix of three polarization directions (x, y, and z) that are directed to the camera using lenses with a finite numerical aperture. As such, the field received by the camera also contains three polarization components that are projected onto the 2D camera plane through the 2 × 2 pixel micropolarizer, resulting in 2 detected polarization components (x and y). This is explicitly taken into account in the simulations as explained by Huijben et al. to enable faithful comparison between simulation and experiment [15].

In the timetraces we observe a steady signal baseline that represents the one-photon photoluminescence (PL) signal from the gold nanosphere (see Figures S6 and S7 in the Supplementary Information) and the camera background. A threshold was applied to identify fluorescence bursts above this baseline and the PSF was obtained by averaging all frames in a single event to maximize the SNR. Several examples of extracted PSFs are presented in Figure 3a. We observe a different PSF and a different polarization signature for each binding event, indicating that the events occur stochastically at different locations on the particle, as expected. Events 1, 2, and 4 exhibit elongated Gaussian PSFs and are therefore likely located near the equator of the nanosphere. Additionally, their emissions are significantly polarized, with DoLP values exceeding 0.5. In contrast, event 3 appears to originate from an emitter bound closer to a polar position (between 0° and 45°), exhibiting a doughnut-shaped PSF and a lower DoLP of 0.2. For a 9-nucleotide complementary DNA imager, a typical fluorescent burst lasts <10 frames (1 s) as dictated by the affinity of the imager strand for the docking strand. Occasionally, we observe events that persist far longer, which likely result from multiple emitters simultaneously interacting with a single gold nanosphere. These cases tend to show reduced polarization (DoLP < 0.4), and are excluded from further analysis, as our aim is to investigate single emitter interactions with individual nanoparticles.

The DoLP for each event differs and is not correlated to *α*_apparent_, as shown in Figure 3b. The broad distribution of *α*_apparent_ indeed suggests that the DNA imager strands bind at random locations on the surface of the nanoparticle. Despite the isotropic shape of the nanospheres, many events exhibit high DoLP values (>0.6) , indicating polarized emission. From simulations, we expected emitters bound near the poles (0°–45°) to show donut-shaped PSFs and lower DoLP (<0.4) , but we observed few of such events. This is attributed to the use of epi-illumination, which weakly excites emitters at the poles because of reduced near-field enhancement along the optical axis. In fact, it is well known that the near-field enhancement at the poles of a nanospohere is smaller than one due to destructive interference between the incident wave and the near-field [44]. As a result, most of the detected events arise from the equatorial regions, where the near-field is enhanced compared to the poles (see Figure S8 for near-field enhancements).

This is confirmed by the correlation between the DoLP and the event intensity, as shown in Figure 3c. Most of the points are concentrated around DoLP >0.6 , but a clear increase in average DoLP is observed for higher intensities. This is again caused by the anisotropic near-field enhancement around the sphere that results in bright events for equatorial binding. These experimental observations are broadly consistent with the numerical model presented above and with recent findings by Novák et al., who analyzed emission in two polarization channels for Au–Ag nanospheres [37].

**Figure 4:**
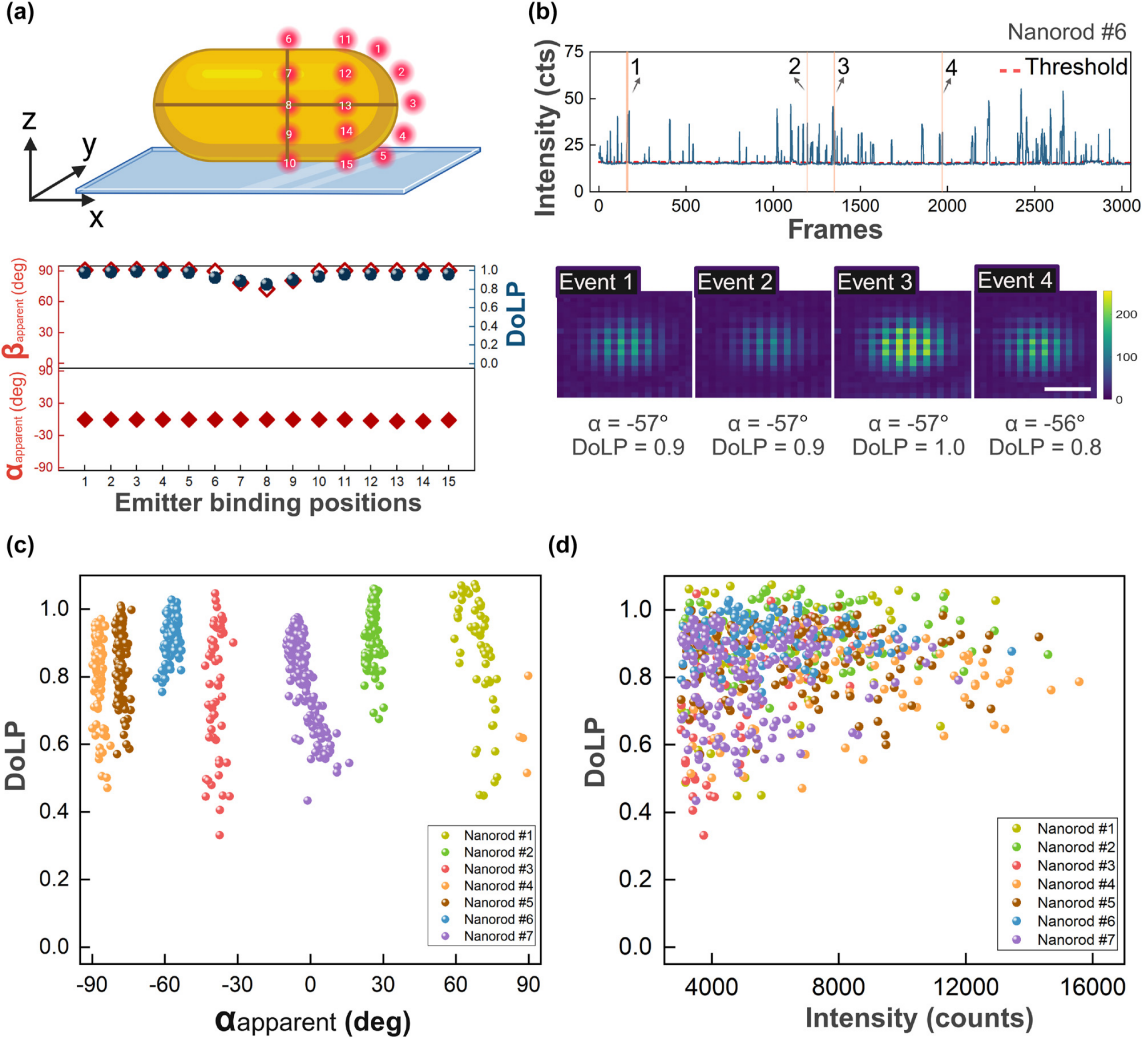
Polarized emission from freely rotating emitters interacting with single nanorods (average diameter 40 nm diameter, average LSPR 700 nm). (a) Simulations of emitters (675 nm emission) were performed at 15 different positions relative to the nanorods. The angles *α*_apparent_ and *β*_apparent_, as well as DoLP were extracted from the simulated PSFs. (b) Intensity time trace from a DNA-PAINT experiment on single gold nanorods, together with four PSFs extracted by averaging all photons within a single binding event. Scale bar 200 nm. (c) Correlation between *α*_apparent_ and DoLP for 7 individual nanorods. (d) Correlation between DoLP and event intensity (total camera counts per event) for the same 7 particles.

We then investigated the effect of particle anisotropy by employing gold nanorods that are broadly used as e.g. biosensors [45], [46], [47], [48]. We first simulated the polarized PSFs of single emitters near a gold nanorod (40 nm wide, 92 nm long) with a longitudinal plasmon resonance at 700 nm, resonant with the emission of ATTO-655 at 675 nm. Freely rotating ATTO-655 were placed along three regions: the particle’s tips (positions 1–5), along the transverse axis (positions 6–10), and the intermediate positions (11–15), as shown at the top of Figure 4a. The simulations in Figure 4a depict that the in-plane polarization angle consistently and strongly aligns with the longitudinal plasmon mode, parallel to the long axis of the nanorod, regardless of the location of the emitter. The DoLP remains close to 1 for most positions, indicating strong polarization along the nanorod. The exception is position 8 which is right at the intersection of the short and long axes, where the DoLP drops to 0.8, likely due to partial coupling to the transverse-plasmon mode. Similarly, the out-of-plane polarization angle (*β*_apparent_) stays close to 90° for most positions. These results suggest that single-emitter emission near gold nanorods is strongly polarized along the long axis of the nanorod due to coupling of the emitter to the dipolar longitudinal plasmon resonance (see Supplementary Figures S9 and S10 for more details).

Zuo et al. observed similar behavior for the in-plane angle of emission [36], but they were unable to extract the DoLP and Stokes parameters because the emission was only detected in two orthogonal polarization channels.

We then performed DNA-PAINT experiments where we employed DNA-functionalized gold nanorods [49] with an ensemble-averaged width of 40 nm and an average LSPR at 700 nm. In the case of nanorods, the one-photon photoluminescence signal is also strongly polarized because it is enhanced by the longitudinal plasmon [50]. Therefore, the particle’s one-photon photoluminescence was subtracted to enable faithful polarimetry of plasmon-enhanced fluorescence signals as shown in Figure 4b. For experimental data from nanorods, we refer to the Supplementary Information. In contrast to the measurements on spheres, and in agreement with the numerical simulations, we now observe that the emission is highly polarized and unidirectional for all events. The extracted angle *α*_apparent_ represents the orientation of the nanorods relative to the substrate [34], [35], [51]. The scatter plot in Figure 4c depicts the correlation between the DoLP and *α*_apparent_ for seven distinct nanorods on the same substrate. Each nanorod is represented by a different color, highlighting their unique orientations by the narrow distribution of *α*_apparent_.

The general trend shows that DoLP remains high, with an average value above 0.8, which matches the simulations. This suggests that the coupling between the emitters and the plasmonic modes of the nanorods results in polarized emissions. However, there is still a noticeable spread in DoLP values even for the same nanoparticle. This is partly attributed to the limited brightness of some events, as shown in Figure 4d. Here we observe a weak correlation between the event brightness and the DoLP, where higher DoLP is observed for brighter events that likely originate from tip-mediated binding.

For GNR, the SNR has an average of 43 ± 16, corresponding to a relative DoLP error of approximately 3 %. For GNS, the average SNR is 26 ± 11, yielding a relative DoLP error of about 5 % (with the simple approximation *σ*_DoLP_/DoLP ≈ 1/SNR). At these SNR levels, the bias in DoLP is minimal, and the dominant contribution to the uncertainty arises from the relative error.

**Figure 5:**
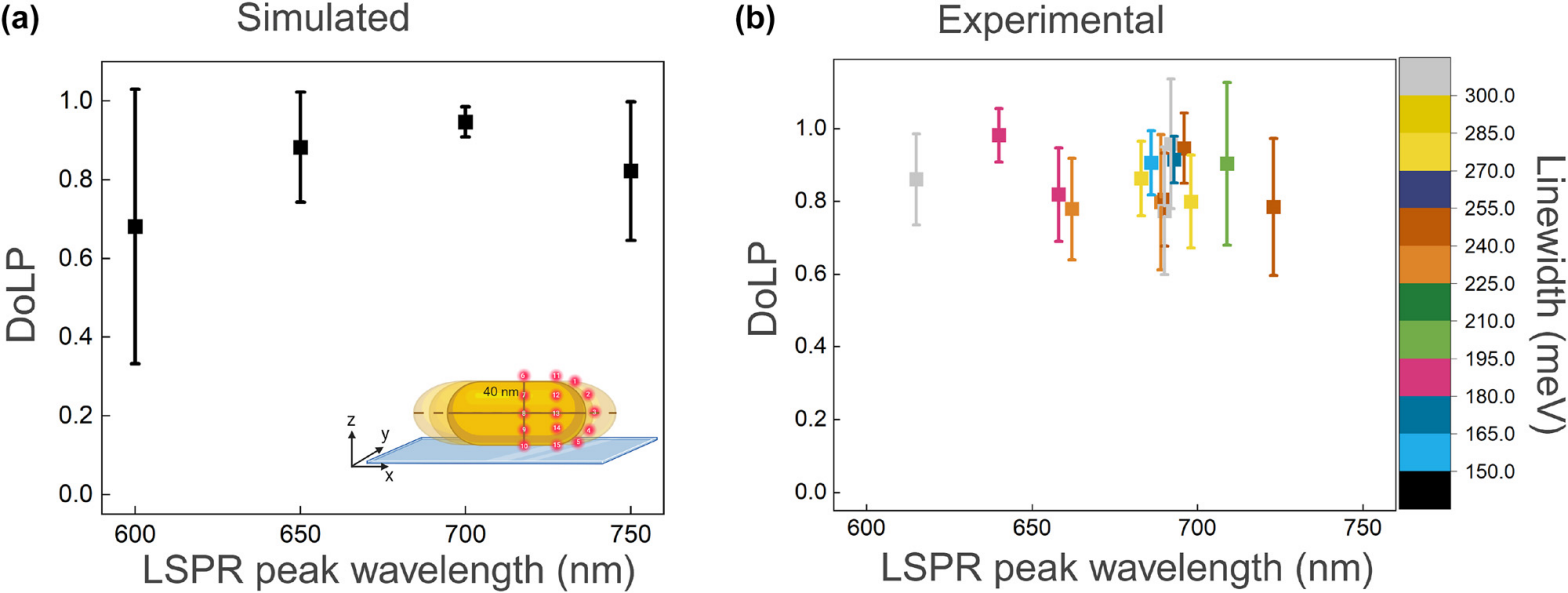
Correlation of DoLP with LSPR peak wavelength. (a) Simulated results showing the relationship between DoLP and the peak LSPR wavelength for nanorods with varying aspect ratios. Emitters are placed at different positions for each nanorod size, as illustrated in the inset. (b) Experimental results depicting the correlation between DoLP, peak LSPR wavelength, and linewidth of single nanorods.

Factors such as nanorod size, LSPR wavelength and linewidth may influence the observed spread in DoLP. To quantify these effects, we first simulate the DoLP for nanorods with varying aspect ratios. As shown in Figure 5a, the simulations indicate that the DoLP is maximized when the LSPR of the nanorod resonantly couples with the emission of the dye. The underlying reason for this is that dipolar coupling between the emitter and the plasmon is strongest in resonant conditions. As a result, the majority of detected photons are those emitted by the plasmon, resulting in a polarization that is dominated by the plasmon’s dipolar response with a unity DoLP.

This trend is confirmed experimentally, where we measured the single-particle scattering spectra to extract both plasmon wavelength and linewidth (see Figure S11 in the Supplementary Information). The results are shown in Figure 5. As in the simulations, we do not observe an obvious trend between the average DoLP and the LSPR peak wavelength: the DoLP remains relatively high (mostly >0.8 ) in the LSPR peak wavelength range from 620 to 720 nm. This suggests that neither the LSPR peak wavelength nor linewidth strongly affects the DoLP because the emission is dominated by the plasmon in all cases investigated here. Surprisingly, we do not observe the increase in the spread of DoLP that the simulations predict for shorter and longer aspect ratios. We hypothesize that this is caused by the algorithm that selects events with SNR > 10 only, thereby discarding events caused by a fluorophore that is only weakly coupled to the LSPR.

## 3 Conclusions

In summary, we present a straightforward method to quantify polarized emission from single fluorophores coupled to plasmonic particles. The method is affordable and does not require addditional optics in the detection path. FDTD simulations were performed on gold nanospheres and nanorods to obtain polarized PSFs from freely rotating emitter binding to the nanoparticles at different positions. The Stokes parameters for polarized PSFs were simulated to determine the dipolar angles (*α* and *β*) and DoLP for the coupled system. Simulation results indicated that emitters binding close to the equator of a gold nanosphere gave the highest DoLP and dipolar emission is confined in the x-y plane or the detector plane while the DoLP decreases to 0 for emitters binding near the poles of a nanosphere as the dipolar emission is perpendicular to the detector plane. The results also demonstrated that the dipolar angle of an emitter coincides with its binding location on the nanosphere, though it is confined to one-quarter of the sphere’s surface area. In contrast, the dipolar emission from emitters coupled nanorods was polarized in the direction of the longitudinal axis with high DoLP, regardless of the emitter’s position on the nanorod.

We perform DNA-PAINT experiments on DNA-coated nanospheres (100 nm in diameter) and nanorods (40 nm diameter, 700 nm LSPR) to obtain polarized PSFs using a polarization camera. Fluorescent events were observed when DNA imager strands (with dye ATTO 655) transiently bound to the nanoparticles, resulting in polarized emission. The distribution of in-plane dipolar emission and DoLP is broad for nanospheres while nanorods have a narrower distribution for *α* and DoLP as they align with the longitudinal plasmon mode. The experimental results validate the simulated polarized emission results.

## 4 Methods

### 4.1 Lumerical FDTD simulations

The electromagnetic fields from a dipole source near a gold nanoparticle are numerically computed in Ansys Lumerical using the finite-difference time-domain (FDTD) method. A nanoparticle (sphere or rod) is placed on a glass coverslip with a refractive index (RI) of 1.52, surrounded by a medium with RI of 1.33. The simulation window has dimensions of 8 µm × 8 µm × 320 nm with a mesh size of 2 nm in a region of 200 nm × 200 nm × 180 nm around the nanoparticle. The simulation time is set to 40 fs, and the dielectric function of the nanoparticle is taken from Johnson and Christy [52]. The dipole source emits at a wavelength of 675 nm (representing ATTO-655 used in the experiments) and is located 4 nm from the nanoparticle’s surface [53].

A near-field detector, located 20 nm below the water-glass boundary, measures the electromagnetic fields in the simulation domain. These near-fields are projected into the far-field using the ‘farfieldexact’ function, which represents the light’s angular distribution, similar to a microscope’s back focal plane. The full details of the FDTD Lumerical simulations are available in our previous work [15].

### 4.2 Single-emitter PSF simulation

The calculated far-fields (**E**), comprising *E*_*x*_, *E*_*y*_, and *E*_*z*_, are imported into a custom MATLAB script to calculate the point spread function of the emitter on the polarization camera. To achieve this, we calculate the Debye–Wolf diffraction integral to focus the fields onto the camera plane [15]. The polarization camera is a regular CMOS camera with a grid of polarizers in front of the chip. The grid of polarizers creates groups of 4 pixels (2 × 2) that resolve polarization angles at 0°, 45°, 90°, and 135°. To determine the intensity on each polarized pixel, we first apply a Jones matrix (*M*) for a linear polarizer with a transmission axis at *η* that matches the transmission axis of the respective polarization mask at the pixel. The electric fields (**E′**) for each pixel were then extracted using:

(9) $$E^{\prime }=ME,$$

where *M* is the Jones matrix for a linear polarizer under angle *η* given by

(10) $$M=\left(\begin{array}{cc} \cos^{2}\eta & \sin\eta \cos\eta \\ \sin\eta \cos\eta & \sin^{2}\eta \end{array}\right).$$

The transmission axis *η* represents the orientation of the polarizer within each pixel and can take values 0°, 45°, 90°, and 135° (shown in Figure 1). We used [135°, 0°, 90°, 45°] combination of pixel orientation to calculate (**E′**). Finally, the intensity (I) was calculated in the image plane as

(11) $$I=|E_{x}^{\prime }{|}^{2}+|E_{y}^{\prime }{|}^{2}.$$

The contribution from the z-component of the **E′** is not taken into account as it is two orders of magnitude lower than the $E_{x}^{\prime }$ and $E_{y}^{\prime }$ components. The intensities from three perpendicular dipole emitter orientations were added without a weighing factor to simulate a freely rotating dye that rotates faster than the fluorescence rate.

### 4.3 Sample preparation

First, glass coverslips (22 mm × 22 mm; thickness #1.5) were sonicated in a methanol bath for 20 min and dried with nitrogen gas. The coverslips were then hydrophilized by ozone cleaning for 60 min. The coverslips were then dip coated in a solution of MPTMS (5 % v/v) in ethanol (purity > 99.9 %) for 3 min, followed by ethanol rinsing and nitrogen drying for thiolation.

We obtained CTAB-coated 100 nm nanospheres from Nanoseedz (product NS-100-50). To get a 2× concentrated solution of gold nanosphere, 500 µL of stock solution was centrifuged at 1,500 rcf for 3 min, and the pellet was re-dispersed in 250 µL of 1 mM CTAB in Milli-Q water. Then 50 µL of concentrated nanosphere solution was drop-casted on the thiolated coverslips and spin-coated at 2,000 rpm for 1 min. The coverslips with nanospheres were generously rinsed with methanol, phosphate-buffered saline (PBS), and water to remove the residual CTAB and weakly bound particles. For nanorods (A12-40-700-CTAB, from NanoPartz) sample preparation, 200 µL of stock solution was centrifuged for 3 min at 2,400 rcf in a vial and then re-suspended in 200 µL of 1 mM CTAB in Milli-Q water. The rest of the preparation was performed in the same manner as for the nanospheres.

The gold nanoparticles were then incubated with a mixture of 5 µM single-stranded DNA docking strands (5′ SH-CAT CAT CAT ACG CTT CCA ATA ATA CAT CTA-3′) purchased from Integrated DNA Technologies and 1 mM tris(2-carboxyethyl)phosphine hydrochloride in citrate buffer (10 mM, pH 3, 1 M NaCl) for surface functionalization. After 2 h, the slides were rinsed with PBS and buffer B (5 mM Tris-HCl, 10 mM MgCl_2_, 1 mM EDTA, pH 8.0) to remove free DNA strands. The slides were stored in buffer B in a humidity chamber until the optical measurement.

### 4.4 Single-molecule fluorescence microscopy

We employed an inverted single-molecule fluorescence microscope with a polarization camera (DYK 33UX250, The Imaging Source, with a 2,448 × 2,048 resolution and a 3.45 µm × 3.45 µm pixel size). DNA-PAINT measurements were performed on an inverted total internal reflection fluorescence microscope (Nikon) with a 100×/NA 1.49 objective.

The depth of field of an objective lens with NA = 1.49 is approximately 600 nm, which may lead to a minor defocus between emission events occurring at *θ* = 0° and *θ* = 180°. This defocus is explicitly accounted for in the simulations [15], where we observe a negligible effect even for 100 nm spheres. Therefore, it is not considered in the experimental analysis. The effect of detection NA on the polarization properties is described in Figure S13. The prepared samples were inserted in a fluidic cell to enable buffer exchange. DNA-PAINT imaging was done using 400 pM imager strands (3′ − *ATTO*655 − *TAT GTA GAT C* − 5′; from Integrated DNA Technologies) labeled with ATTO655 in buffer B (5 mM Tris-HCl, 10 mM MgCl_2_, 1 mM EDTA, pH 8.0, filtered). A 637 nm fiber-coupled excitation laser (OBIS FP 637 LX, Coherent) was collimated by a Thorlabs F810APC-635 and spectrally cleaned using a clean-up filter (Thorlabs BP FLH635-10). The sample was illuminated in an epi-illumination configuration with circularly polarized light. The power density in the sample plane was 5 kW/cm^2^ for the nanospheres sample and 2 kW/cm^2^ for the nanorods. A long-pass filter (Thorlabs FELH0650) in the detection path removed residual excitation and Rayleigh-scattered light. Fluorescence time traces were recorded in an uncompressed format at 10 fps using the IC Capture software and further processed using ImageJ, Matlab, and Python.

To extract the PSF of each single-molecule binding event in the DNA-PAINT experiment, we process the fluorescence time traces of a single particle as follows. We plot a time trace by calculating the average camera count in an ROI of 20 × 20 pixels centered on a nanoparticle. We then extract the binding events by defining a threshold by first running a median filter, after which an offset was added to dynamically account for baseline variations. The PL signal was subtracted from each averaged fluorescent event by using the average signal below the threshold in the nearby frames to extract the fluorescent signal of a single emitter. Finally, we averaged all frames that belonged to the same event to obtain its PSF in an ROI of 20 × 20 pixels.

## Supplementary Material

Supplementary Material Details
