# Supplementary material for: Single-shot Stokes polarimetry of plasmon-coupled single-molecule fluorescence
Source: Nanophotonics. 2025 Nov 6;14(27):5105–16. doi: 10.1515/nanoph-2025-0352 (PMC12717926; doi:10.1515/nanoph-2025-0352)
Supplement: Supplementary file 1 — Supplementary Material Details [file j_nanoph-2025-0352_suppl_001.pdf]

Supplementary Information

Single-shot Stokes Polarimetry of Plasmon-Coupled Single-Molecule Fluorescence

Sarojini Mahajan[†], Yuyang Wang[†], Teun A.P.M. Huijben[‡], Rodolphe Marie[‡], Peter Zijlstra[*,†]

†*Department of Applied Physics and Institute for Complex Molecular Systems, Eindhoven University of Technology, 5600 MB Eindhoven, The Netherlands*

‡*Department of Health Technology, Technical University of Denmark (DTU), 2800 Kongens Lyngby, Denmark*

E-mail: p.zijlstra@tue.nl

Contents

S1: Fixed dipole emitter on a glass substrate 2
S2: Polarized emission from a fixed emitter on a GNS 3
S3: Polychromatic effects on polarized emission near GNS 4
S4: SNR impact on polarized emission from a single emitter on a GNS 5
S5: polarization distortion due to dichroic mirror (DC) 7
S6: Experimental characterization of single gold nanosphere 9
S7: Polarized events (PSFs) from emitters binding to a gold nanosphere 10
S8: Simulated electric field enhancement around GNS and GNR 11
S9: Polarized emission from a fixed emitter on a GNR-along curvature 12
S10: Polarized emission from a fixed emitter on a GNR-along short axis 13
S11: Experimental characterization of a single gold nanorod 14
S12: Polarized emission PSFs from emitters binding to a gold nanorod 15
S13: Impact of objective NA on polarization measurements 16

S1 Fixed dipole emitter on a glass substrate

We simulated fixed dipole emitters, both in-plane and out-of-plane, on a glass substrate using the FDTD method in Lumerical. The emitter was set to an emission wavelength of 675 nm and positioned 5 nm above the glass surface, which had a refractive index of 1.52. The surrounding medium had an RI of 1.33. Detailed simulation parameters are provided in the Methods section. From the simulated point spread functions (PSFs) exported as TIFF files for various dipole orientations, we extracted the in-plane angle (α), out-of-plane angle (β), and the degree of linear polarization (DoLP).

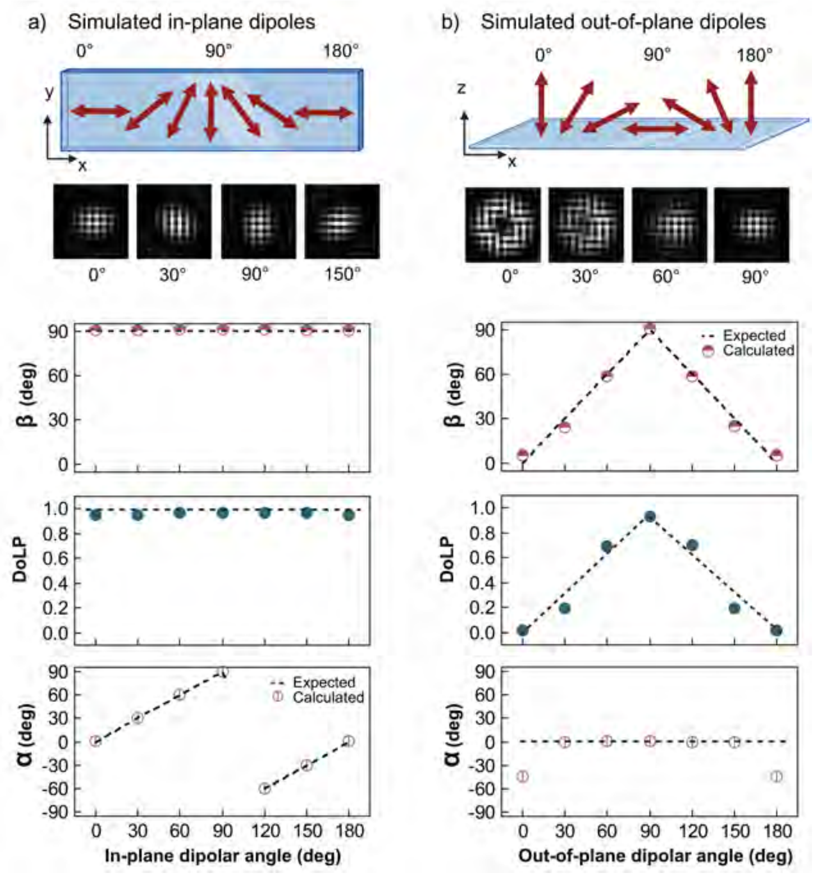

Figure S1: Polarized emission from a fixed dipole emitter on a glass substrate. (a) Schematic of in-plane dipole emitters rotated from 0° (aligned with the x-axis) to 90° (aligned with the y-axis) in 30° increments. The graphs below show the out-of-plane angle (β), degree of linear polarization (DoLP), and in-plane angle (α) as functions of the dipole orientation in the substrate plane. The simulated dipole orientations align well with the calculated in-plane angle α (red symbols). (b) Schematic of out-of-plane dipole emitters rotated from 0° (aligned with the z-axis) to 90° (aligned with the x-axis) in 30° increments. The calculated β values match the simulated orientations, as shown in the graph. The DoLP trend closely follows β, reflecting their proportional relationship. The in-plane angle α remains 0° for all orientations except when the dipole is aligned with the z-axis, where α is undefined and assigned a value of 45°.

S2 Polarized emission from a fixed emitter on a GNS

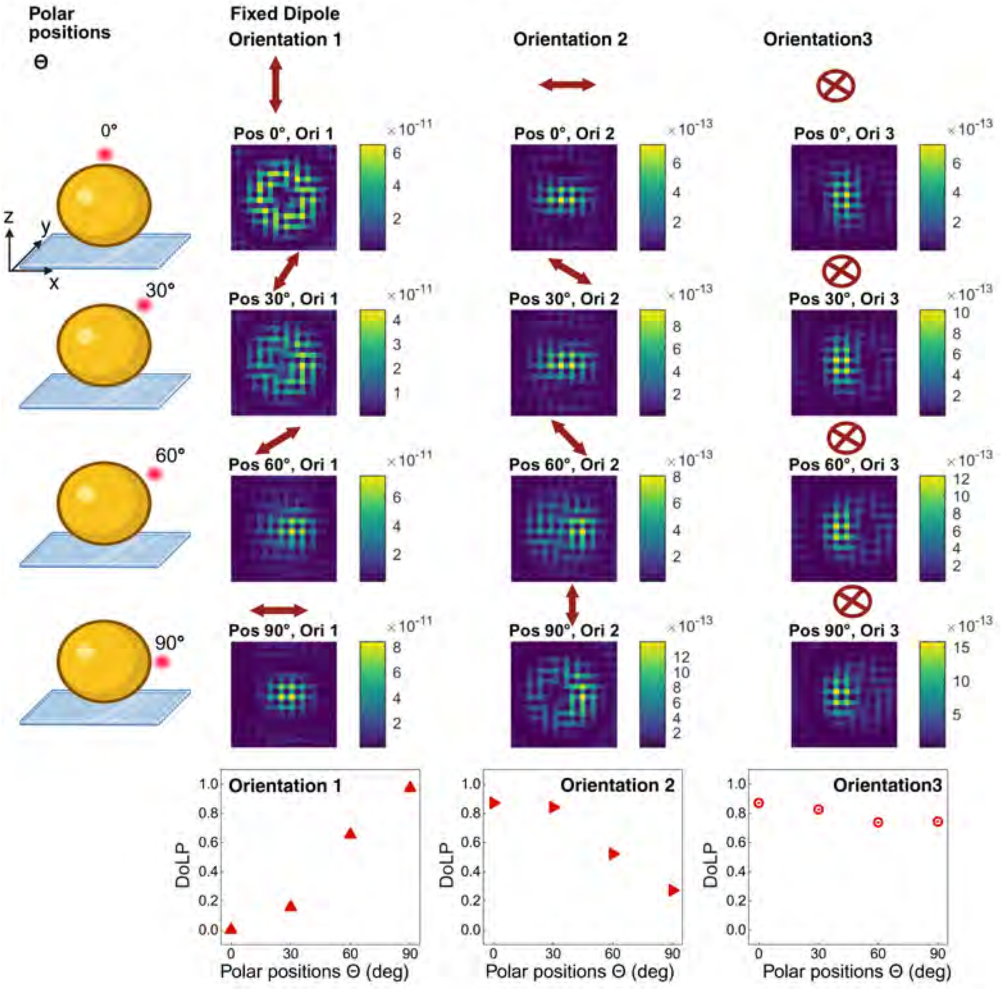

Figure S2: Polarized emission patterns from a fixed dipole emitter positioned at different polar angles (Θ) on a gold nanosphere, shown for three mutually perpendicular dipole orientations. Orientation 1 is radial, pointing outward from the sphere's center. Orientation 2 is tangential to the sphere's surface. Orientation 3 lies in the x–y plane perpendicular to the other two. The dipole oriented radially (Orientation 1) produces the strongest point spread function (PSF) intensity compared to the tangential and x-y plane orientations. The degree of linear polarization (DoLP) measured for a freely rotating dipole, modeled as the sum of the three orthogonal orientations, closely follows the trend of orientation 1, indicating that the freely rotating dipole behavior is dominated by the radial component. This indicates that a freely rotating dipole near a gold nanosphere behaves similarly to a fixed dipole oriented radially relative to the NP surface.

S3 Polychromatic effects on polarized emission near GNS

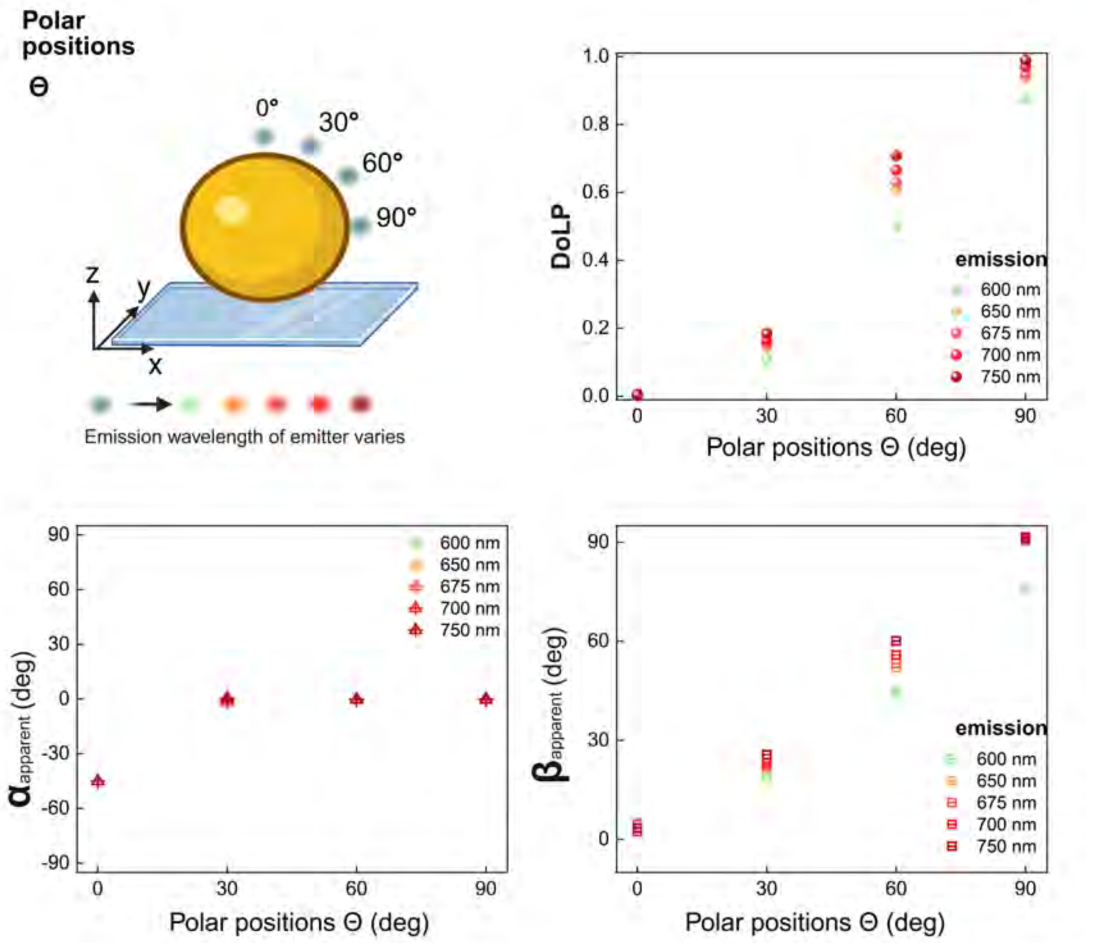

Figure S3: Emission-wavelength-dependent analysis of polarized emission from a freely rotating dipole emitter across different polar angles (Θ). **(Top left)** Schematic showing the emitter positioned at various polar angles, simulated at five different emission wavelengths. **(Top right)** Degree of linear polarization (DoLP) as a function of polar angle for emission wavelengths ranging from 600–750 nm. As the emission wavelength approaches the plasmon resonance of the GNS, both DoLP and the apparent out-of-plane angle (β_{apparent}) decrease by approximately 10–15%, depicted by a green symbol. **(Bottom left)** In-plane angle (α_{apparent}) shows negligible impact due to polychromatic emission of an emitter. The minimal wavelength-dependent variation observed for wavelengths above 650 nm supports the validity of using a single-wavelength approximation (675 nm) for modeling emitter behavior in a polychromatic experimental setting (with a 650 nm long-pass filter).

S4 SNR impact on polarized emission from a single emitter on a GNS

To simulate noise in our system, the noiseless point spread function (PSF) image was first normalized to the range [0, 1], and then scaled to achieve target signal-to-noise ratios (SNR) of 1, 4, 8, 16, and 32. For each SNR level, shot noise was added by sampling each pixel from a Poisson distribution, with the mean determined by the scaled image intensity. Subsequently, Gaussian noise was added to model camera noise, based on dark noise data obtained from an image captured in complete darkness (i.e., without any illumination). The resulting images were then rescaled to the 8-bit range [0, 255] to match the output format of a polarization-sensitive camera, enabling further analysis as shown in Figure S4.
Figure S4 illustrates the effect of the signal-to-noise ratio (SNR) on the extraction of the polarization parameters of the emitters at various polar angles ($\theta = 0°, 30°, 60°, 90°$). Raw polarization images at different SNR levels (1–32) demonstrate improved image clarity with increasing SNR. The corresponding plots show that the DoLP (in blue), β_{apparent} (red open diamonds), and α_{apparent} (red filled diamonds) become more accurate and precise as the noise decreases. At low signal-to-noise, the pixel values are dominated by noise and all of similar value, resulting in a DoLP and β that are close to zero and a broadly distributed extracted value for α. For example, at $\theta = 0°$ we observe that α_{apparent} remains highly uncertain independent of SNR (as shown in the top right plot). This is due to the undefined nature of the in-plane polarized emission at this angle, as illustrated in Figure S1. These results highlight the importance of having an SNR>10 for reliable single-molecule polarimetry. In the remainder of this report, we therefore discard any single-molecule events with a SNR<10. Since DoLP and β_{apparent} exhibit similar trends, we exclude β_{apparent} from further data visualization.

To estimate the photon budget in our experiments, we analysed the fluorescence time trace shown in Figure 3 in the main text. A typical fluorescent event yields an average of ∼40 camera counts per pixel per frame. After subtracting the background 1PL signal and camera baseline (∼20 counts), the net signal is ∼20 counts per pixel per frame. Let us consider a scenario with completely polarized emission at 90°. Considering the 20×20 pixel region of interest defined by the PSF and 2×2 polarization segmentation, there are 100 pixels per polarizer orientation. Pixels with a 90° polarizer receive $20 \times 100 = 2000$ counts, while pixels at 45° and 135° each receive $0.5 \times 20 \times 100 = 1000$, totaling $2 \times 1000 = 2000$ counts. Pixels at 0° receive zero counts. Altogether, this sums to 4000 counts per frame per event or 40,000 counts per second. Due to a lack of exact data, we assume typical camera specifications with a gain of 0.25 e^- per ADU and quantum efficiency (QE) of 30%. This translates to approximately 10,000 incident photons per second, yielding an estimated SNR of ∼100 for these events. Some events may have a signal-to-noise ratio (SNR) below 10, e.g., due to a shorter binding time. We exclude these events (15% of total events) from our analysis because the extracted values of DoLP are unreliable, as shown in Figure S4. This places our experimental regime well above the low-SNR threshold, indicating that the accuracy of DoLP and angular emission parameters for the selected events is not limited by photon budget.

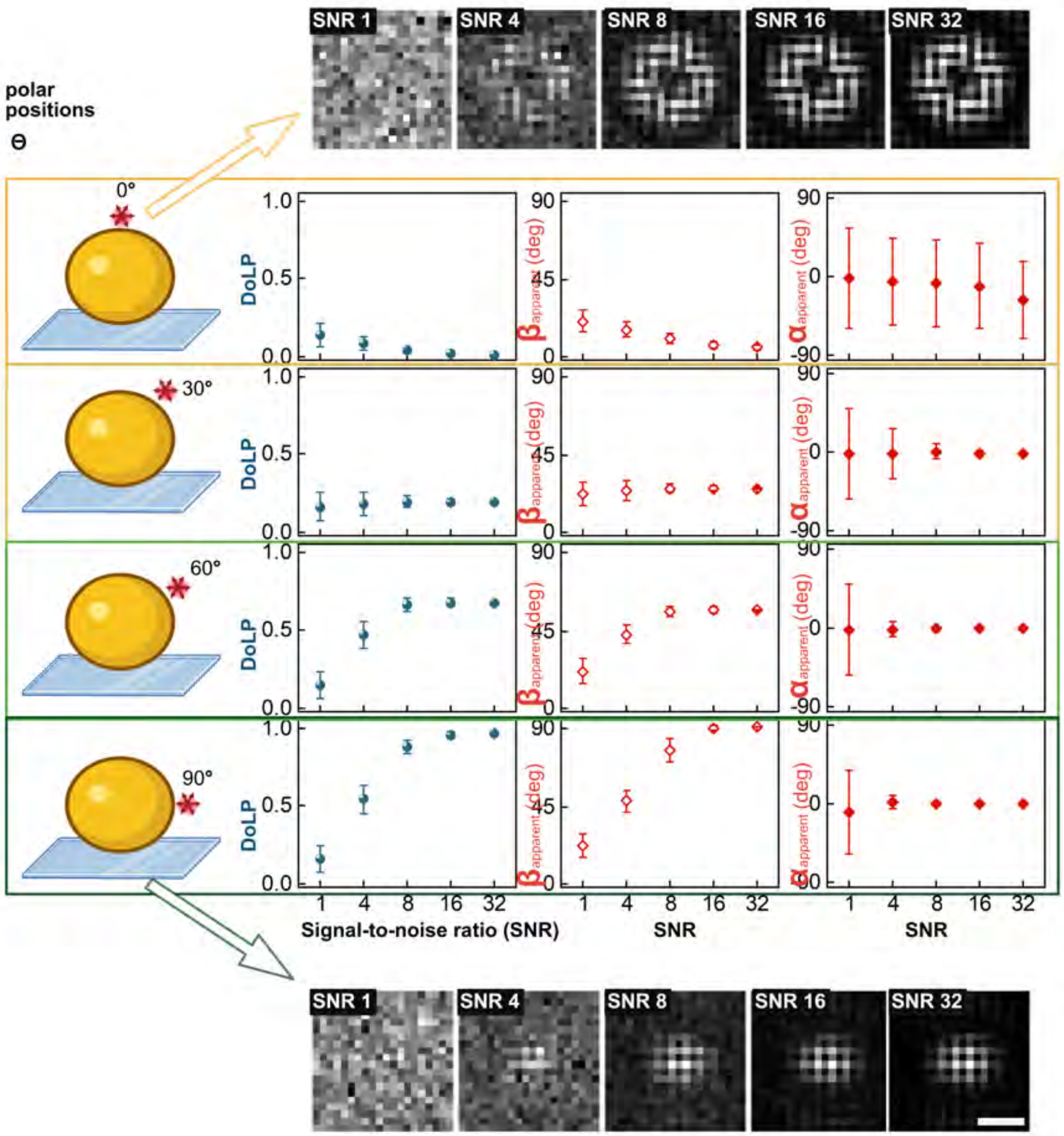

Figure S4: Influence of signal-to-noise ratio (SNR) on polarization parameters of emitted light at varying polar angles on a gold nanosphere($\Theta = 0°$, $30°$, $60°$, $90°$). The top and bottom rows show raw polarization images at different SNR levels (1 to 32) for $\Theta = 0°$ and $90°$, respectively. The three columns of plots in each row represent the extracted values of Degree of Linear Polarization (DoLP, blue), out-of-plane polarized emission β_{apparent} (red open diamonds), and in-plane polarized emission α_{apparent} (red filled diamonds) as a function of SNR. Error bars indicate uncertainty in parameter estimation. As SNR increases, the accuracy and precision of DoLP, β_{apparent}, and α_{apparent} improve across all polar orientations. Scale bar in bottom right image denotes 200 nm and applies to all images.

S5 polarization distortion due to dichroic mirror (DC)

To evaluate the degree of polarization distortion introduced by the optical setup, we illuminated the polarization-sensitive camera with the beam from a white LED source that was passed through a linear polarizer. The polarizer was rotated to obtain a controlled linear polarization and extract the Stokes parameters for various polarization angles. Particularly, the dichoric beamsplitter that is used for laser in-coupling may introduce polarization distortion because the Fresnel reflection coefficients of the mirror become polarization dependent under 45 degrees incidence. We therefore compared the Stokes parameters without any beamsplitter, a dichroic beamsplitter, and a 50:50 beamsplitter. The results are plotted in Figure S5c, where we show the extracted Stokes parameters for these three scenarios. Note that we inserted an emission filter around 675 nm in the detection path to enable quantification of the polarization distortion at the emission wavelength of ATTO655.
In the absence of any beam splitter, the Stokes parameters S_1 and S_2 follow a cosine pattern as expected.[1] When S_1 is at its maximum, indicating that the light is horizontally polarized (0°) or vertically (90°), S_2 is zero. Meanwhile, S_0, the sum of the intensities of all pixels, remains nearly constant, as expected from equation (3) in the Methods section. However, when a dichroic mirror is introduced into the optical path, the calculated Stokes parameter exhibits anomalous behavior: S_1 no longer follows the expected cosine-squared function, indicating that the dichroic mirror induces polarization-altering effects of the horizontal and vertical polarization components. However, when the dichroic mirror is replaced with a 50:50 beam splitter, the expected cosine-squared behavior is restored, as illustrated in Figure S5c.

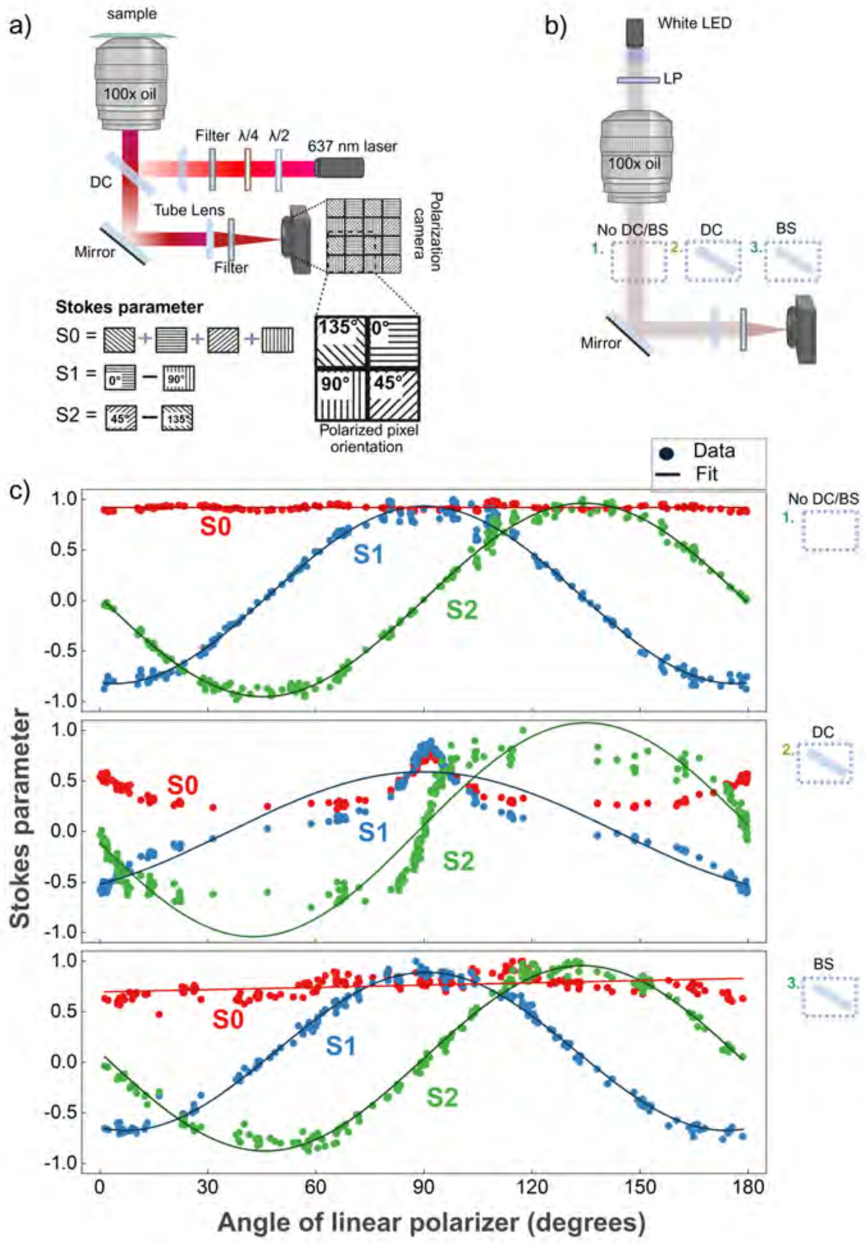

Figure S5: a) Schematic of a standard fluorescence microscopy setup with a polarization camera, where a circularly polarized laser, using a half-wave plate ($\lambda/2$) and a quarter-waveplate ($\lambda/4$), excites the sample, and the emitted light is collected using a micropolarizer array (transmission axis at 0°, 45°, 90°, or 135°) integration into the camera. The Stokes parameter can be calculated from the intensity value of each micropolarizer pixel. b) Experimental setup to evaluate DC-induced polarization distortion. A polarized LED is imaged in three cases: 1. without DC/BS, 2. with a DC, and 3. with a beamsplitter (BS). c) Stokes parameters (S_0, S_1, S_2) plotted against the micropolarizer polarization axis for all three conditions. The solid line in the first graph illustrates the oscillatory nature of S_1 and S_2, showing their cosine-based variation.

S6 Experimental characterization of single gold nanosphere

After the DNA-PAINT measurements on nanospheres, the setup was switched to perform dark-field scattering spectroscopy for single-particle determination. The red laser was replaced with a supercontinuum white-light source (SuperK Compact from NKT Photonics), and all wavelength filters in the detection path were removed. The microscope was aligned for TIRF illumination, and a beam block was placed below the objective to block direct light from the source. Scattering signals were collected from 500 to 750 nm with a 10 nm bandwidth, scanning the same sample area.

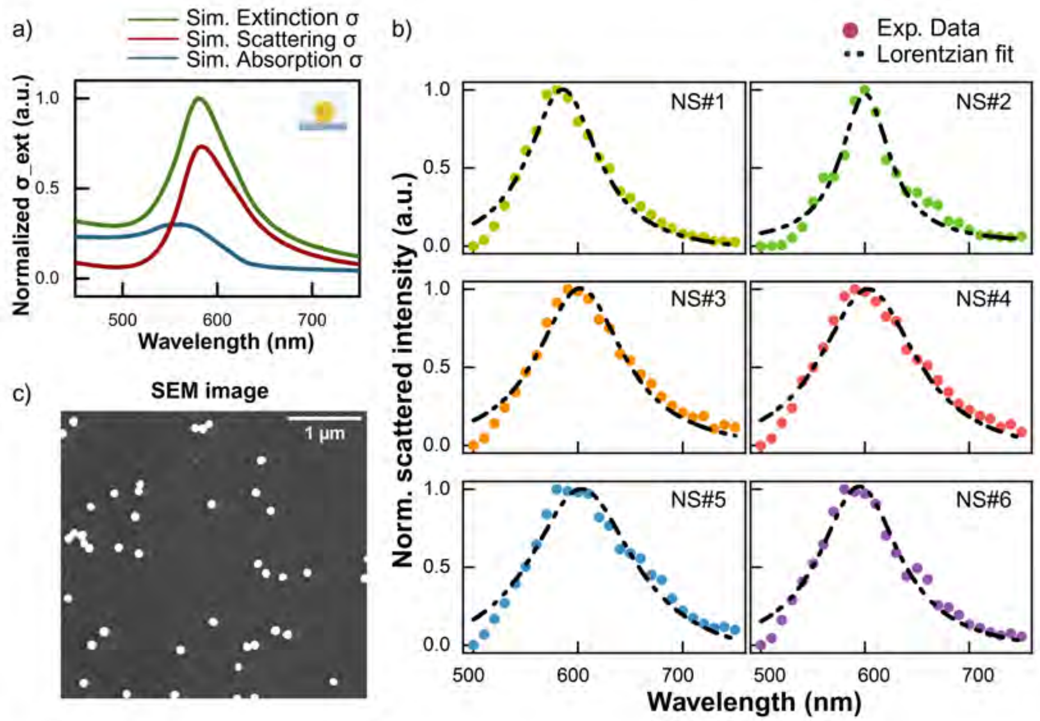

Figure S6: Dark-field scattering spectroscopy for characterization of single gold nanoparticles. a) Simulated extinction spectrum of a single 100 nm diameter gold nanosphere on a glass substrate immersed in water. The plasmon resonance peak is centered at 580 nm with a linewidth of approximately 180 meV. b) Experimental scattering spectra of individual gold nanospheres functionalized with DNA strands on glass. Data points represent normalized scattering intensity measured over wavelengths from 500 to 750 nm. A Lorentzian fit is applied to extract the plasmon resonance peak position and spectral linewidth. The observed SPR peaks range from 580 to 605 nm, with linewidths between 190 and 400 meV.[2] c) SEM image of the sample surface showing well-dispersed individual nanospheres, confirming that the sample preparation method avoids clustering.

S7 Polarized events (PSFs) from emitters binding to a gold nanosphere

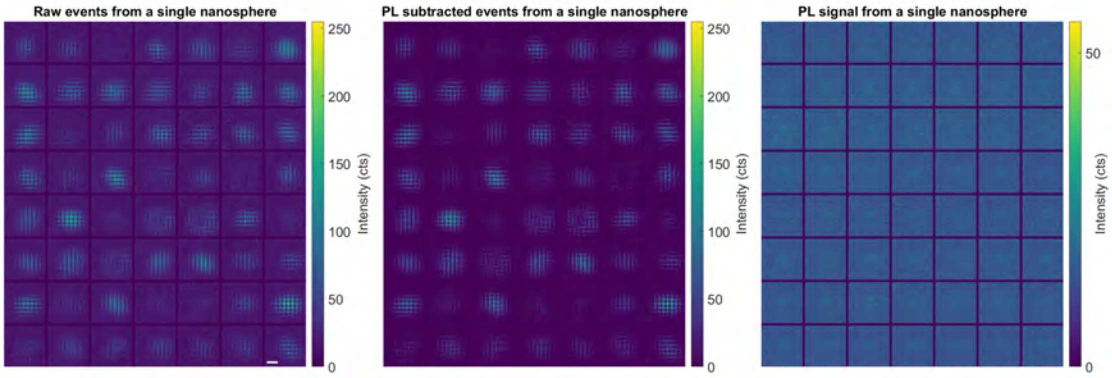

Figure S7: Experimental raw frames capturing imager DNA binding to docking DNA on a gold nanosphere on a polarization-sensitive camera. The exposure was set at 10 fps and 1 pixel in the image is 35 nm. The middle panel displays photoluminescence (PL)-subtracted frames highlighting the binding events, which were used to analyze polarized emission from single emitters on the nanosphere. The last panel shows the one-photon PL signal from the same nanosphere. The PL signal is weak because the excitation wavelength (637 nm) is off-resonance with the gold nanosphere's peak plasmon resonance around 580 nm.[3]

S8 Simulated electric field enhancement around GNS and GNR

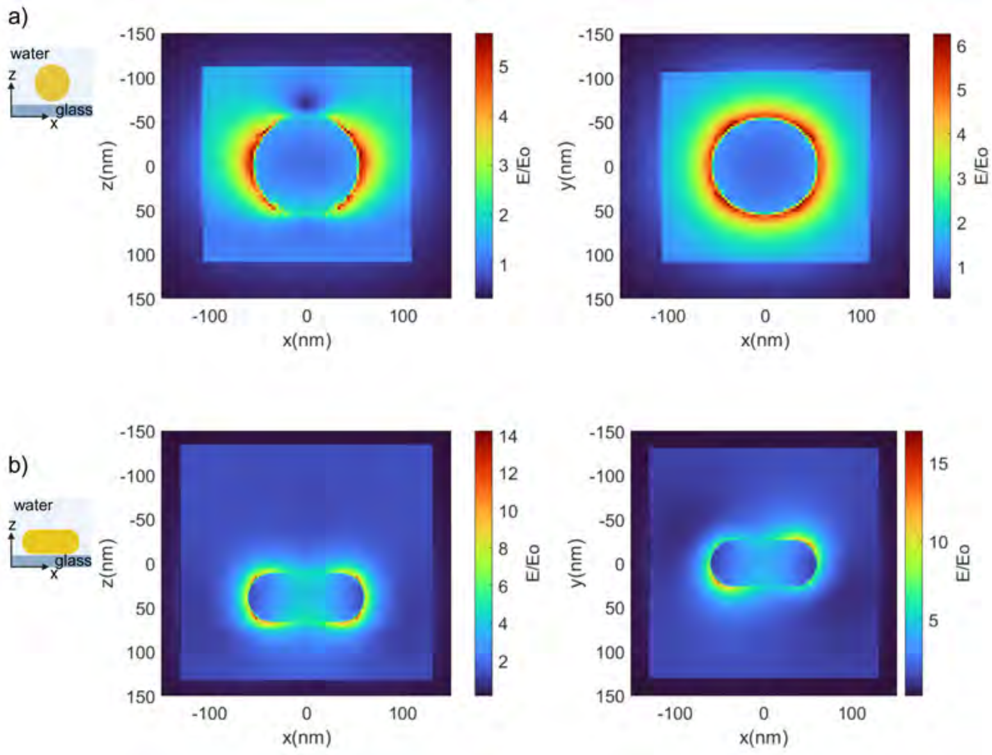

Figure S8: a) Electric field distribution for a 100-nm gold nanosphere on a glass substrate immersed in water, illuminated with circularly polarized light at an off-resonance wavelength of 637 nm under epi-illumination. The left and right panels show cross-sectional field enhancement ($|E|/|E_0|$) in the x-z and x-y planes, respectively. In the x-y plane, the near-field distribution is symmetric due to the spherical geometry. In the x-z plane, field enhancement at the poles is reduced below one due to destructive interference between the incident and scattered fields.[4] b) Electric field distribution for a gold nanorod (40 nm $\times$ 92 nm) under similar conditions, excited closer to its longitudinal plasmon resonance at 700 nm. The left and right panels show cross-sections in the x-z and x-y planes, respectively. Strong field enhancement is observed at the nanorod tips due to excitation of the longitudinal plasmon mode. In the x-y plane, the field distribution is slightly asymmetric and tilted toward the short axis due to off-resonant excitation and coupling to the transverse mode.

S9 Polarized emission from a fixed emitter on a GNR-along curvature

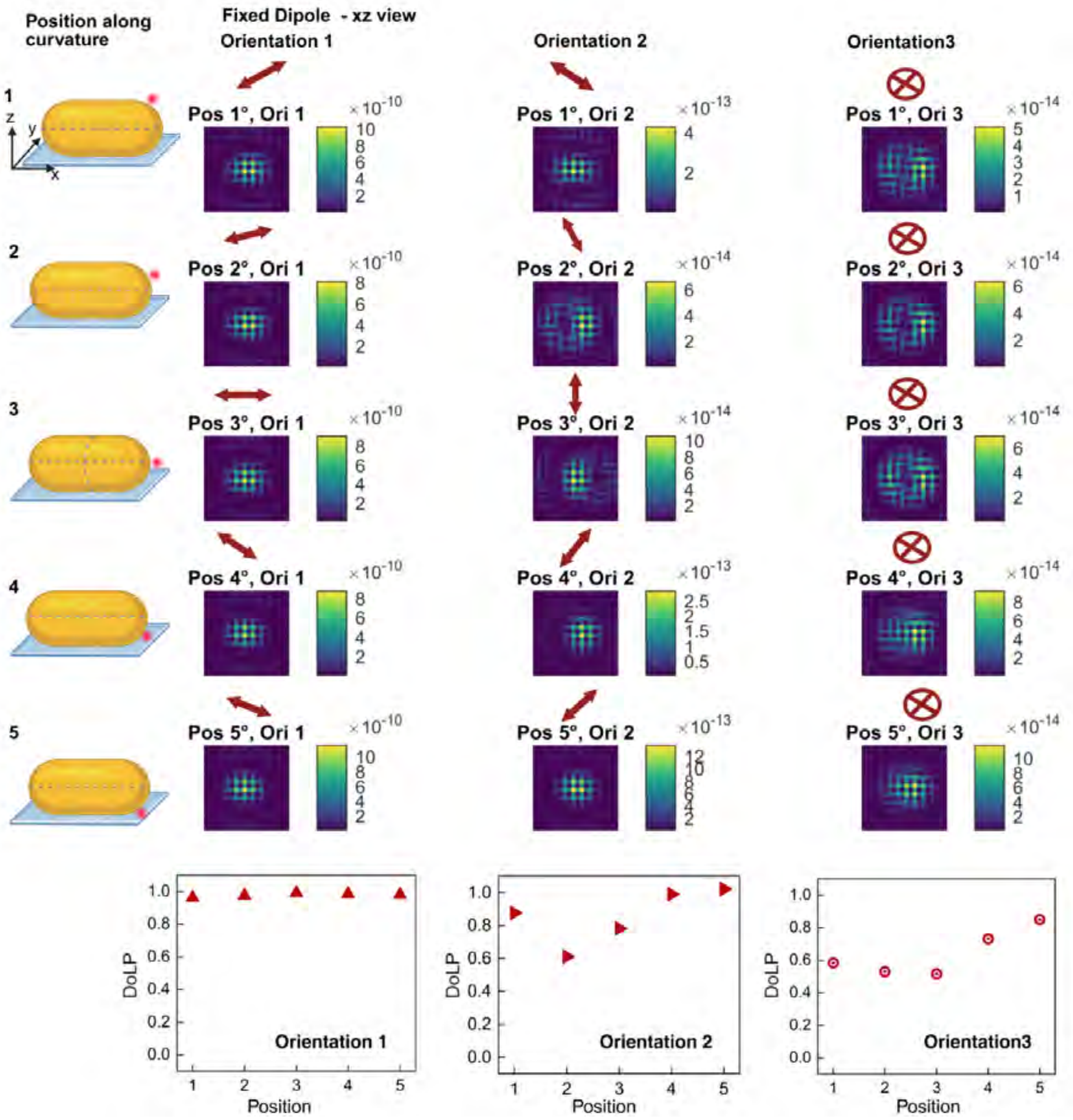

Figure S9: Polarized emission patterns from a fixed dipole emitter positioned at different positions on a gold nanorod along the curvature, shown for three mutually perpendicular dipole orientations. Orientation 1 is radial, pointing outward from the curvature center. Orientation 2 is tangential to the curvature's surface. Orientation 3 lies in the x-y plane perpendicular to the other two. A dipole oriented radially (Orientation 1) produces the strongest point spread function (PSF) intensity, compared to tangential or in-plane (x–y) orientations, similar to the response observed for gold nanospheres. The DoLP for Orientation 1 remains close to unity across all positions. In the case of a freely rotating dipole, the contribution from Orientation 1 dominates due to the stronger induced dipole moment in the radial direction.

S10 Polarized emission from a fixed emitter on a GNR- along short axis

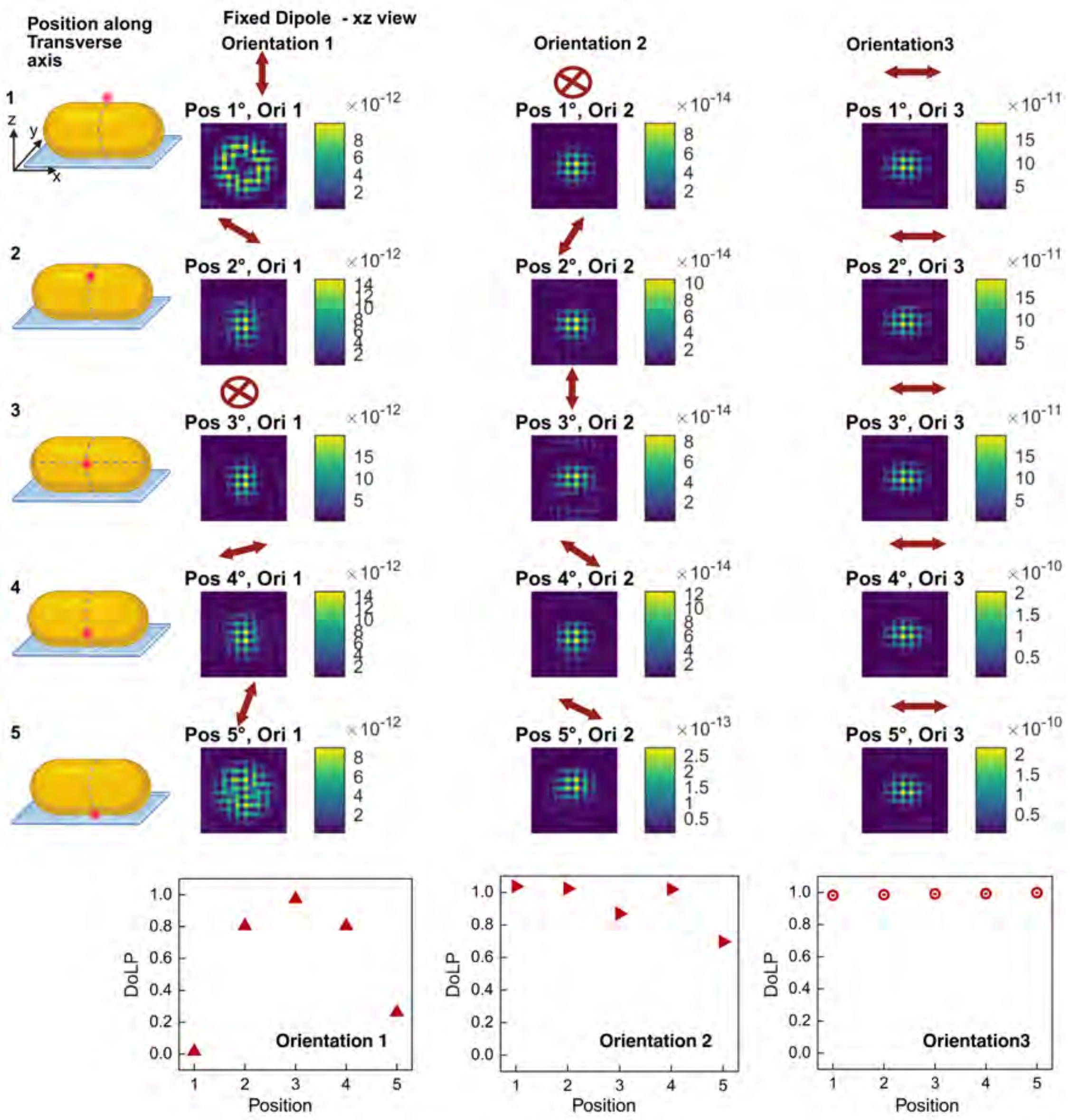

Figure S10: Three mutually perpendicular dipole orientations are placed at different positions on a gold nanorod short axis curvature. Orientation 1 is radial, pointing outward from the curvature center. Orientation 2 is tangential to the curvature's surface. Orientation 3 lies in the x-y plane parallel to x-axis. In these simulations, we observe stronger polarized PSFs for Orientation 3, which corresponds to the dipole aligned along the longitudinal plasmon mode. However, Orientation 1 also contributes significantly in a freely rotating dipole, as the transverse mode influences both DoLP and PSF intensity. For position 3 and orientation 1, we clearly see that induced dipole leads to strongly polarized emission with DoLP close to unity.

S11 Experimental characterization of a single gold nanorod

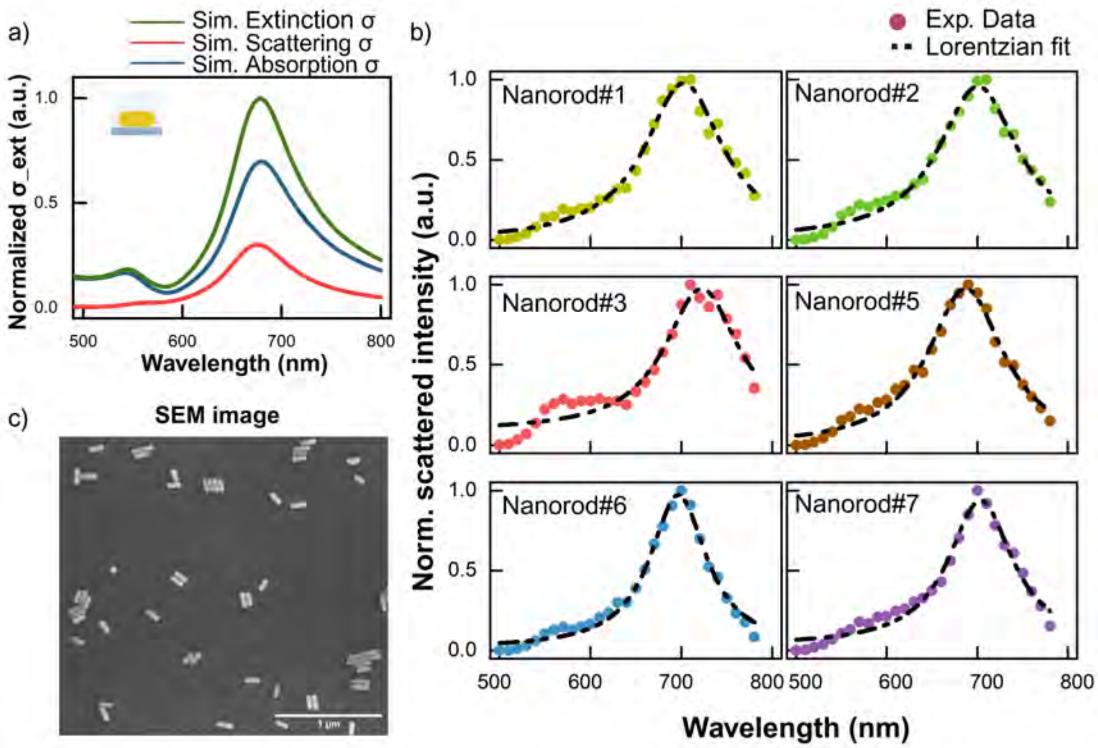

Figure S11: Dark-field scattering spectroscopy for characterization of single gold nanorods. a) Simulated extinction spectrum of a single gold nanorod with dimensions 40 nm × 92 nm on a glass substrate immersed in water. Two distinct plasmon resonance peaks are observed: the transverse mode around 550 nm and the longitudinal mode near 690 nm. b) Experimental scattering spectra of individual gold nanorods functionalized with DNA strands on a glass substrate. Data points represent normalized scattering intensity measured over the 500–780 nm range. A Lorentzian fit is applied to extract the resonance peak and spectral linewidth. The observed longitudinal SPR peaks are centered around 700 nm with linewidths ranging from 200 to 250 meV.[2] c) SEM image of the sample surface showing well-dispersed single nanorods, confirming that the sample preparation yields sparsely spaced individual particles. The scale bar represents 1 µm.

S12 Polarized emission PSFs from emitters binding to a gold nanorod

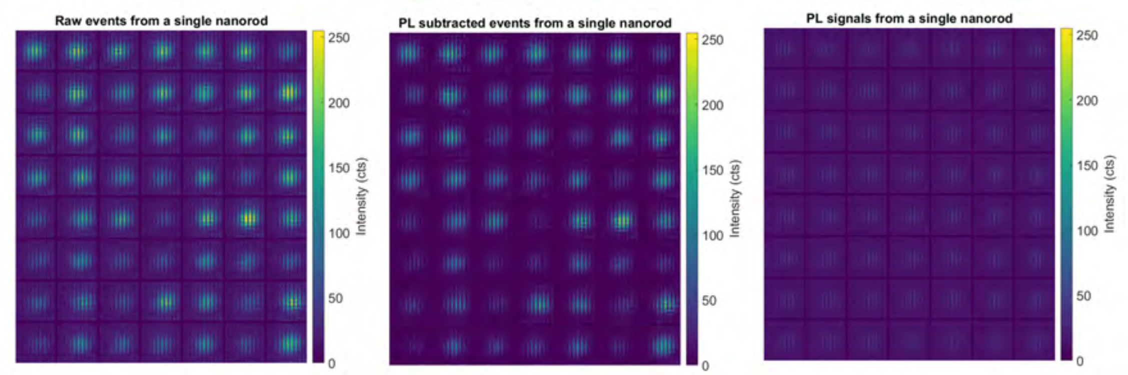

Figure S12: Experimental raw frames capturing imager DNA binding to docking DNA on a gold nanosphere recoroded on a polarization-sensitive camera. The exposure was set at 10 fps and 1 pixel in the image is 35 nm. The events appear similar for all the frames as the polarized emission is the direction of nanorod orientation on the glass substrate. The middle panel displays photoluminescence (PL)-subtracted frames highlighting the binding events, which were used to analyze polarized emission from single emitters on the nanorods. The last panel shows the one-photon PL signal from the same nanorod. The PL signal is stronger than the PL signal from the nanosphere as the excitation wavelength (637 nm) is on-resonance with the longitudinal plasmon mode which is around 700 nm. The resemblence of PL subtracted events from the emitters and PL signal from the nanorods is a strong indicator that the emission polarization is dominated by the longitudinal plasmon mode.

S13 Impact of objective NA on polarization measurements

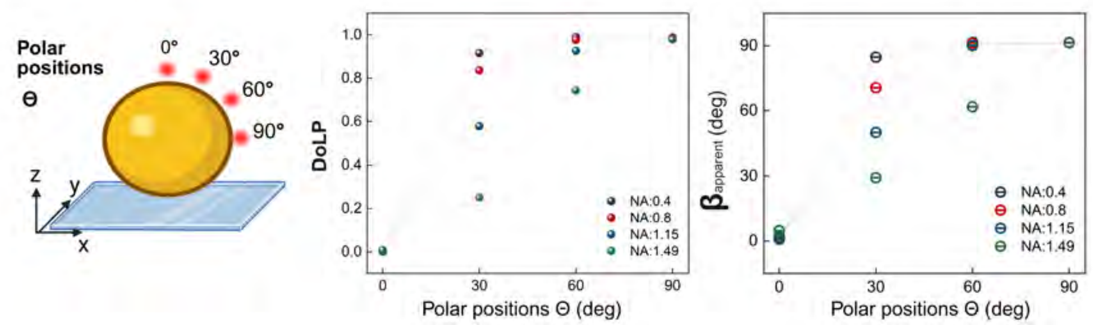

Figure S13: Impact of numerical aperture (NA) on the Degree of Linear Polarization (DoLP) and the apparent out-of-plane angle (β_{apparent}). For lower NAs (0.4 and 0.8, water immersion), the DoLP saturates across all polar positions except at the top pole, where it approaches zero. For NA = 1.15, β_{apparent} is overestimated and saturates for polar positions beyond $\sim 60°$. In contrast, the apparent in-plane angle (α_{apparent}) remains unaffected across all NA values and is therefore not shown. Overall, these results demonstrate that while high-NA collection introduces a small depolarization, its influence on the extracted parameters is negligible in the context of our experiments.

References

(1) Lin, Y.-H.; Huang, H.-H.; Wang, Y.-J.; Hsieh, H.-A.; Chen, P.-L. Image-based polarization detection and material recognition. *Optics Express* **2022**, *30*, 39234–39243.

(2) Sönnichsen, C.; Franzl, T.; Wilk, T.; von Plessen, G.; Feldmann, J.; Wilson, O.; Mulvaney, P. Drastic reduction of plasmon damping in gold nanorods. *Physical review letters* **2002**, *88*, 077402.

(3) Hu, H.; Duan, H.; Yang, J. K.; Shen, Z. X. Plasmon-modulated photoluminescence of individual gold nanostructures. *Acs Nano* **2012**, *6*, 10147–10155.

(4) Novotny, L.; Hecht, B. *Principles of nano-optics*; Cambridge university press, 2012.
